# Fluid balance after cardiac arrest: Any impact on outcome? Insights from the MIMIC IV database

**DOI:** 10.1016/j.resplu.2025.101085

**Published:** 2025-09-06

**Authors:** Songsong Luo, Xiaoyuan Shen

**Affiliations:** Department of Critical Care Medicine, The First People’s Hospital of Xiaoshan District, Xiaoshan Affiliated Hospital of Wenzhou Medical University, Hangzhou 311200 Zhejiang, China

Dear Editor,

We read with great interest the recent study by Didier et al. (2025),^1^ which examined the association between early cumulative fluid balance and mortality in patients with post–cardiac arrest shock, utilizing data from the MIMIC-IV database. The authors addressed a clinically relevant question and applied multivariable models to support their conclusions. While the study offers important insights into a potentially modifiable aspect of post-resuscitation management, several methodological considerations may warrant further discussion.

First**,** the proportion of diuretic use was significantly higher among day-3 survivors than non-survivors (62.5 % vs 19.7 %, *P* < 0.001), yet this variable was excluded from the multivariable model. Diuretic therapy not only affects urine output and fluid balance but may also indicate more stable hemodynamics, preserved renal function, and better prognosis.^2^ Without adjusting for this factor, the observed association between positive fluid balance and mortality could be overestimated. Future analyses could include diuretic use both presence and standardized dose alongside renal function and hemodynamic parameters, to differentiate intentional fluid removal from oliguria due to renal injury.

Second, both maximum vasoactive inotropic score (VIS) and cumulative fluid balance were reported as independent predictors of mortality. Given that high VIS often reflects severe circulatory failure, which may prompt more aggressive fluid resuscitation and reduce urine output, substantial confounding or interaction is possible.^3^ Without collinearity checks, VIS × fluid balance interaction tests, or stratified analyses by VIS level, the independent effect of fluid balance may be overestimated. Incorporating interaction terms and stratified models could clarify whether the prognostic role of fluid balance varies with shock severity.

Third**,** the study entered age as a categorical rather than continuous variable (median 64 years). Older patients may have more comorbidities, impaired renal function, and different fluid strategies, potentially modifying the fluid balance mortality association.^4^ Retaining age as a continuous variable, and modeling with restricted cubic splines or generalized additive models, could reveal non-linear relationships and avoid arbitrary cut-offs.

In conclusion, this study provides valuable evidence linking positive fluid balance to early mortality in post-CA shock. Nevertheless, adjusting for key treatment-related confounders, testing for major interactions, and using advanced modeling approaches could strengthen causal inference and inform individualized fluid management in this high-risk population.

## Declaration of competing interest

The authors declare that they have no known competing financial interests or personal relationships that could have appeared to influence the work reported in this paper.
